# Prediction of Long-Term Recovery From Disability Using Hemoglobin-Based Models: Results From a Cohort of 1,392 Patients Undergoing Spine Surgery

**DOI:** 10.3389/fsurg.2022.850342

**Published:** 2022-03-16

**Authors:** Matteo Briguglio, Paolo Perazzo, Francesco Langella, Tiziano Crespi, Elena De Vecchi, Patrizia Riso, Marisa Porrini, Laura Scaramuzzo, Roberto Bassani, Marco Brayda-Bruno, Giuseppe Banfi, Pedro Berjano

**Affiliations:** ^1^IRCCS Orthopedic Institute Galeazzi, Scientific Direction, Milan, Italy; ^2^IRCCS Orthopedic Institute Galeazzi, Intensive Care Unit, Milan, Italy; ^3^IRCCS Orthopedic Institute Galeazzi, GSpine 4, Milan, Italy; ^4^IRCCS Orthopedic Institute Galeazzi, Laboratory of Clinical Chemistry and Microbiology, Milan, Italy; ^5^University of Milan, Department of Food, Environmental and Nutritional Sciences, Division of Human Nutrition, Milan, Italy; ^6^IRCCS Orthopedic Institute Galeazzi, Spine Unit 1, Milan, Italy; ^7^IRCCS Orthopedic Institute Galeazzi, Spine Unit 2, Milan, Italy; ^8^IRCCS Orthopedic Institute Galeazzi, Spine Unit 3, Milan, Italy; ^9^Vita-Salute San Raffaele University, Faculty of Medicine and Surgery, Milan, Italy

**Keywords:** preoperative care, orthopedic procedures, vertebral column, hemoglobin, anemia, patient-reported outcome measures, enhanced recovery after surgery, spine surgery

## Abstract

Hemoglobin and its associated blood values are important laboratory biomarkers that mirror the strength of constitution of patients undergoing spine surgery. Along with the clinical determinants available during the preadmission visit, it is important to explore their potential for predicting clinical success from the patient's perspective in order to make the pre-admission visit more patient-centered. We analyzed data from 1,392 patients with spine deformity, disc disease, or spondylolisthesis enrolled between 2016 and 2019 in our institutional Spine Registry. Patient-reported outcome measure at 17 months after surgery was referred to the Oswestry disability index. High preoperative hemoglobin was found to be the strongest biochemical determinant of clinical success along with high red blood cells count, while low baseline disability, prolonged hospitalization, and long surgical times were associated with poor recovery. The neural network model of these predictors showed a fair diagnostic performance, having an area under the curve of 0.726 and a sensitivity of 86.79%. However, the specificity of the model was 15.15%, thus providing to be unreliable in forecasting poor patient-reported outcomes. In conclusion, preoperative hemoglobin may be one of the key biomarkers on which to build appropriate predictive models of long-term recovery after spine surgery, but it is necessary to include multidimensional variables in the models to increase the reliability at the patient's level.

## Introduction

Spinal disorders are common and the prevalence increases in the aging population (1). In view of the recent advancements in spine treatments, surgery is considered clinically effective from a medical perspective. However, healthcare systems are progressively moving toward value-based business models (2), with the definition of clinical success increasingly based on patient-reported outcome measures (PROMs) (3). The Oswestry disability index (ODI) is a widely-used, validated, and self-administered questionnaire to assess a patient's functional impairment related to the spinal condition, encompassing questions about personal care, movements, sleeping, and social life (4), and can be used to evaluate the outcomes from the patient's perspective (5). The advent of PROMs calls for more patient-centric healthcare, which outlines the need for early preoperative pathways. Identifying the determinants that affect the individual's daily activities in the long term can help maintain high-quality standards (6). One of the parameters known to mirror the functional reservoirs required for proper recovery is hemoglobin (7–9). An abnormal circulating level before surgery is considered a risk factor for poor medical and surgical outcomes in spinal patients (10), also being a predictor of mortality in the most severe conditions (11). Hemoglobin is an assembly of four globular polypeptide chains that fill the warp of red blood cells, carrying up to four oxygen molecules attached to iron atoms. Together with the erythrocytes and their volume over total blood (i.e., hematocrit), hemoglobin reflects oxygen carrying capacity, functional iron levels, and correct erythropoiesis (12). Hemoglobin concentration is one of the benchmarks for planning transfusion therapy strategy (13) along with other laboratory parameters such as hematocrit (14). Therefore, preoperative iron optimization is considered a key aspect of patient blood management (15) and enhanced recovery after surgery (16). To the authors' knowledge, there are no studies in spine surgery that have investigated the potential of preoperative hemoglobin in predicting clinical success from a patient's point of view. We studied a large cohort of patients undergoing spine surgery for deformities, disc disease, and other back conditions to identify predictors of long-term functional status using hemoglobin-based models.

## Materials and Methods

### Study Population

The research included patients enrolled in the institutional Spine Registry (SpineReg; ClinicalTrials.gov number: NCT03644407), which is a prospective observational registry recruiting patients undergoing spine surgery incepted in 2015 by our hospital IRCCS Orthopedic Institute Galeazzi of Milan (Italy). To answer the research question, we extracted from the registry the patients who met the following eligibility criteria: ≥18 years of age, enrolment between 2016 and 2019, the presence of at least one ODI assessment at 6-, 12-, or 24-month follow-up. The extraction excluded data of patients with a diagnosis of tumors, admission for complications, and surgical procedures involving the cervical spine. A 30% reduction from baseline was considered as the minimum clinically important difference (MCID) in the ODI score in order to categorize the outcomes (17). A secondary analysis was planned using the raw reduction of 12.7 points as classification threshold, which is more restrictive in categorizing successful surgeries (18). Patients who had a preoperative ODI < 12.7 were excluded from the extraction query. After data extraction and integration with routine parameters, the study sample comprised 1,392 patients with seventeen variables: gender, age, red blood cells (RBCs), hematocrit (Ht), serum concentration of hemoglobin (Hb), mean corpuscular volume (MCV), mean corpuscular hemoglobin (MCH), mean corpuscular hemoglobin concentration (MCH), C-reactive protein (CRP), type of diagnosis, American society of anesthesiologists physical status classification system (ASA), duration of surgery (DS), length of hospital stay (LOS), preoperative ODI (PREOP ODI), 6-month ODI, 12-month ODI, and 24-month ODI. The number of missing values counted 85 CRP, 14 DS, 555 6-month ODI, 422 12-month ODI, and 731 24-month ODI.

### Data Handling

The simple imputation technique of the last observation carried forward (LOCF) was used to include patients with incomplete 24-month ODI, with the last reported scores being used in place of the missing values. The months of follow-up were then weighed on the imputations, thus obtaining the ODI scores at 17 months (661 24-month ODI + 576 12-month ODI + 155 6-month ODI). In Table 1 is reported the structure of the dataset. Subsequently, three new variables were imputed. The first variable was the difference between the baseline ODI and the last ODI at 17 months (ΔODI PRE vs. last 17). The second variable was the presence or absence (1,0) of clinical improvement at 17 months (17-month progress 30%), calculated as a 30% decrease from baseline ODI to the last ODI at 17 months. The third variable was the presence or absence (1,0) of clinical improvement at 17 months (17-month progress 12.7), calculated as a raw reduction in the ODI score≥12.7 from baseline ODI to the last ODI at 17 months. Two new sets classified the sample by gender code (males = 0, females = 1) and diagnosis (spine deformities: 4, disc diseases: 5; back surgeries: 6). Specifically, the group of spine deformities included kyphosis and scoliosis, and the group of back surgeries included spondylosis, spondylolisthesis, stenosis, and elective treatment of fractures. The dataset was explored for what concerned the presence of outliers among the primary variables, and a new dataset for regression analysis was created after the elimination of outliers. The following number of outliers were excluded based on the interquartile range rule of three: 4 RBCs, 27 MCV, 36 MCH, 2 MCHC, 96 CRP, 2 DS, and 21 LOS.

**Table 1:** Structure and missing data of the study group dataset.

**Captions**	**Cases *n***	**Missing *n* (%)**
Age (years, continuous)	1,392	0
Gender (dichotomous)	1,392	0
RBCs (10^6^/μl, continuous)	1,392	0
Hematocrit (%, continuous)	1,392	0
Hemoglobin (g/dl, continuous)	1,392	0
MCV (fl/cell, continuous)	1,392	0
MCH (pg/cell, continuous)	1,392	0
MCHC (g/dl, continuous)	1,392	0
CRP (mg/dl, continuous)	1,307	85 (6.11%)
Diagnosis (categorical)	1,392	0
ASA (ordinal)	1,392	0
DS (minutes, continuous)	1,378	14 (1.01%)
LOS (days, continous)	1,392	0
Baseline ODI (continuous)	1,392	0
6-month ODI (continuous)	837	555 (39.87%)
12-month ODI (continuous)	970	422 (30.32%)
24-month ODI (continuous)	661	731 (52.51%)
LOCF 17-month ODI (continuous)	1,392	0
	**TOTAL**	1,807 (7.77%)

*RBCs, red blood cells; MCV, mean corpuscular volume; MCH, mean corpuscular hemoglobin; MCHC, mean corpuscular hemoglobin concentration; CRP, C-reactive protein; ASA, American Society of Anesthesiologists physical status classification system (1, healthy; 2, mild; 3, severe; 4, life threatening; 5, moribund; 6, brain-dead); DS, duration of surgery; LOS, length of hospital stay; ODI, Oswestry Disability Index, ranging from 0 (no disability) to 100 (maximum disability); LOCF, last observation carried forward*.

### Statistics

The IBM SPSS 22 statistics package was used for all statistical analysis. The descriptive variables were reported as means, standard deviation, minimum and maximum values to be reported in Tables 2, 3 (baseline examination), regardless of the type of distribution. In Table 4 (outcome exploration) it was planned to report the most significant biochemical descriptors against the outcomes. Based on the results from the Shapiro-Wilk test on the dataset without outliers, Ht (*p* = 0.989), Hb (*p* = 0.281), and MCHC (*p* = 0.078) were assumed to have normal distribution. Age (*p* < 0.05), RBCs (*p* < 0.05), MCV (*p* < 0.05), MCH (*p* < 0.05), CRP (*p* < 0.05), DS (*p* < 0.05), LOS (*p* < 0.05), and baseline ODI (*p* < 0.05) were assumed to be skewed. The newly created dataset without outliers was used for running descriptive statistics, whereas the raw dataset was planned to be used for inferential statistics. The existence of a difference in the biochemical parameters between males and females was planned to be investigated through the independent *t*-test or the Mann-Whitney *U*-test for normally or not normally distributed values, respectively, and controlling for the homogeneity of variance by Levene's test for equality of variances (adjusted degree of freedom). The existence, strength, and direction of the association between the biochemical parameters and the demographic variable of age were examined using the Pearson product-moment correlation for continuous normally distributed variables or the Spearman rank-order correlation coefficient for skewed data. Regardless of data distribution, blood values and years of age were planned to be reported in scatter plots against the baseline ODI together with the Pearson's correlation and linear regression coefficients (*unstandardized B*). The difference between males and females in baseline ODI was investigated likewise through the Mann-Whitney *U*-test.

**Table 2:** Baseline demographic and biochemical characteristics of the study group.

**Descriptors**	**Values**	**Cases *n***
Age (years)	56.41 ± 15.24 (18.00; 88.00)	1,392
Gender	567 males, 825 females	1,392
**Biochemistry**		
RBCs (10^6^/μl)	04.76 ± 00.49 (02.50; 06.88)	1,392
Hematocrit (%)	42.31 ± 03.64 (27.20; 54.10)	1,392
Hemoglobin (g/dl)	14.04 ± 01.38 (09.20; 18.90)	1,392
MCV (fl/cell)	89.16 ± 05.69 (54.60; 115.20)	1,392
MCH (pg/cell)	29.58 ± 02.20 (18.00; 37.60)	1,392
MCHC (g/dl)	33.16 ± 01.02 (27.50; 36.50)	1,392
CRP (mg/dl)	00.35 ± 00.67 (00.01; 08.74)	1,307

*RBCs, red blood cells; MCV, mean corpuscular volume; MCH, mean corpuscular hemoglobin; MCHC, mean corpuscular hemoglobin concentration; CRP, C-reactive protein*.

**Table 3:** Baseline surgical descriptors of the study group.

**Descriptors**	**ASA**	**Baseline ODI**	**DS *minutes***	**LOS *days***
Total cases (*n* = 1,392)	01.86 ± 00.57	46.84 ± 16.40	220.01 ± 142.28	04.83 ± 03.61
	(01.00; 03.00)	(13.00; 100.00)	(47.93; 813.80)	(01.00; 48.00)
**Clusters of diagnosis**				
Spine deformities (*n* = 216)	01.95 ± 00.55	45.81 ± 16.26	415.46 ± 146.47	08.50 ± 05.75
	(01.00; 03.00)	(13.00; 82.00)	(113.43; 813.80)	(02.00; 48.00)
Disc diseases (*n* = 628)	01.72 ± 00.56	47.75 ± 16.75	171.41 ± 114.13	03.69 ± 02.17
	(01.00; 03.00)	(13.00; 100.00)	(48.02; 772.32)	(01.00; 16.00)
Other back surgeries (*n* = 548)	01.99 ± 00.57	46.21 ± 16.03	197.91 ± 98.86	04.69 ± 02.80
	(01.00; 03.00)	(13.00; 94.00)	(47.93; 690.03)	(01.00; 27.00)

*ASA, American Society of Anesthesiologists physical status classification system (1, healthy; 2, mild; 3, severe; 4, life threatening; 5, moribund; 6, brain-dead); ODI, Oswestry Disability Index, ranging from 0 (no disability) to 100 (maximum disability); DS, duration of surgery; LOS, length of hospital stay. Spine deformities (kyphosis, scoliosis). Disc diseases (back regions). Back surgeries (spondylosis, spondylolisthesis, stenosis, fractures)*.

**Table 4:** Baseline differences in biochemical parameters according to the long-term recovery.

**Descriptors**	**17-month failure**	**17-month success**	**Between-group *p***
RBCs (10^6^/μl)	4.69 ± 0.50 (2.86; 6.31)	4.79 ± 0.48 (2.50; 6.88)	*p* < 0.01
Hematocrit (%)	41.84 ± 3.95 (30.50; 53.30)	42.48 ± 3.50 (27.20; 54.10)	*p* < 0.01
Hemoglobin (g/dl)	13.81 ± 1.47 (9.70; 18.00)	14.12 ± 1.34 (9.20; 18.90)	*p* < 0.01
MCV (fl/cell)	89.54 ± 6.16 (60.20; 106.60)	89.02 ± 5.52 (54.60; 115.20)	*p* < 0.01
MCH (pg/cell)	29.54 ± 2.35 (19.50; 36.60)	29.59 ± 2.14 (18.00; 37.60)	Not significant
MCHC (g/dl)	32.98 ± 1.02(29.80; 36.30)	33.23 ± 1.01 (27.50; 36.50)	*p* < 0.01
CRP (mg/dl)	0.36 ± 0.60 (00.01; 04.39)	0.34 ± 0.69 (00.01; 08.74)	Not significant

*RBCs, red blood cells; MCV, mean corpuscular volume; MCH, mean corpuscular hemoglobin; MCHC, mean corpuscular hemoglobin concentration; CRP, C-reactive protein. Clinical success, a 30% decrease from preoperative ODI at 17 months after surgery*.

Concerning outcome exploration, the Chi-Square test was used to investigate the differences in gender between outcome groups at 17 months using both 30% reduction and the raw 12.7 points reduction. Similarly, it was planned the Mann-Whitney *U*-test for the years of age and the biochemical parameters, taking into account the result of Levene's test for equality of variances based on median (adjusted degree of freedom). The Chi-Square test was also used to investigate the different outcomes according to ASA, diagnosis, DS, and LOS. Subsequently, the probe of two prediction regression models was planned; the first model (PRE_BIO_) would be based on the seven preoperative biochemical markers, while the second model (PERI_BIODEMCLI_) would have included the biochemical, the demographic (gender and age), and the clinical (diagnosis, ASA, baseline ODI, DS, and LOS) variables. The prediction potential of each of the two models on the last ODI was explored through multiple regression analysis on the dataset without outliers. The models were planned to be based on the stepwise method to serially add the next strongest predictor feasibly removing the previously entered predictor not significant. The most significant predictors would be chosen to report the predictive equations. The Wilcoxon signed-rank test was run to match the regression predicted values from the estimated regression equation with the real values of the dataset. The main predictors of each model were tested for the clinically significant outcomes (1,0) by using binary logistic regression (enter method). Neural Networks (NN) analysis was planned to observe the forecasting outcome as a function of each variable-specific model: PRE_BIO_(NN) and PERI_BIODEMCLI_(NN). Given the non-linear nature of this tool, the authentications between inputs and outputs have been run on the raw dataset through the supervised learning technique of Multilayer Perceptron (MLP) procedure to produce a predictive model for clinical success based on the values of the demographic, biochemical, and clinical predictors. After data whitening (the distributions were rescaled so that the mean was zero and the standard deviation was one), the training sample was set at 70%, the testing sample to track prediction at 20%, and the holdout sample to assess the final NN at 10%. The NN architecture was planned to be based on two hidden layers with Sigmoid activation function (real-valued arguments are transformed to the range 0, 1) and on Softmax activation for the output layer (the vector of real-valued arguments is transformed to a vector whose elements fall in the range 0, 1 and sum to 1). The Receiver Operating Characteristic (ROC) curve evaluated the Area Under the Curve (AUC), the sensitivity (true positive outcome), specificity (true negative outcome), and 1–specificity (false positive rate) of model-specific predictors in three successive run of NN, with the normalized importance of the independent predictors independent being reported for the most significant multivariate model.

## Results

### Preoperative Examination

The demographic and preoperative biochemical variables are reported in Table 2. The study cohort showed a prevalence of about 41% of males and 59% of females, with men showing higher values than females of RBCs (05.02 ± 00.46 10^6^/μl vs. 04.58 ± 00.42 10^6^/μl; *U* = 104,835.00, *Z* = −17.389, *p* < 0.00001), Ht (44.53 ± 03.17% vs. 40.79 ± 03.12%; *equalvariancest*_(1, 390)_ = 21.833, *p* < 0.00001), Hb (14.93 ± 01.20 g/dl vs. 13.43 ± 01.14 g/dl; *equalvariancest*_(1, 390)_ = 23.722, *p* < 0.00001), MCH (29.84 ± 02.15 pg/cell vs. 29.40 ± 02.21 pg/cell; *U* = 182,475.00, *Z* = −5.633, *p* < 0.00001), and MCHC (33.53 ± 00.99 g/dl vs. 32.91 ± 00.96 g/dl; *equalvariancest*_(1, 388)_ = 11.909, *p* < 0.00001). No sex differences for MCV (88.98 ± 05.67 fl/cell in males vs. 89.28 ± 05.71 fl/cell in females; *U* = 219,466.00, *Z* = −0.742, *p* = 0.458) and CRP (00.34 ± 00.70 mg/dl in males vs. 00.35 ± 00.65 mg/dl in females; *U* = 173,124.00, *Z* = −0.665, *p* = 0.506).

Males had a mean age of 55.15 ± 15.63 years old and females had 57.28 ± 14.92 years old. A negative association was found between age and RBCs (*Rho* = −00.184, *p* < 0.00001), Ht (*Rho* = −00.116, *p* = 0.00001), Hb (*Rho* = −0.164, *p* < 0.00001), and MCHC (*Rho* = −00.207, *p* < 0.00001). Conversely, age positively associated with MCV (*Rho* = +00.203, *p* < 0.00001), MCH (*Rho* = +00.068, *p* = 0.012), and CRP (*Rho* = +00.198, *p* < 0.00001). The variables concerning surgical and anesthetic parameters are reported in Table 3, indicating data for each of the three clusters of spine diagnosis.

**Figure 1:**
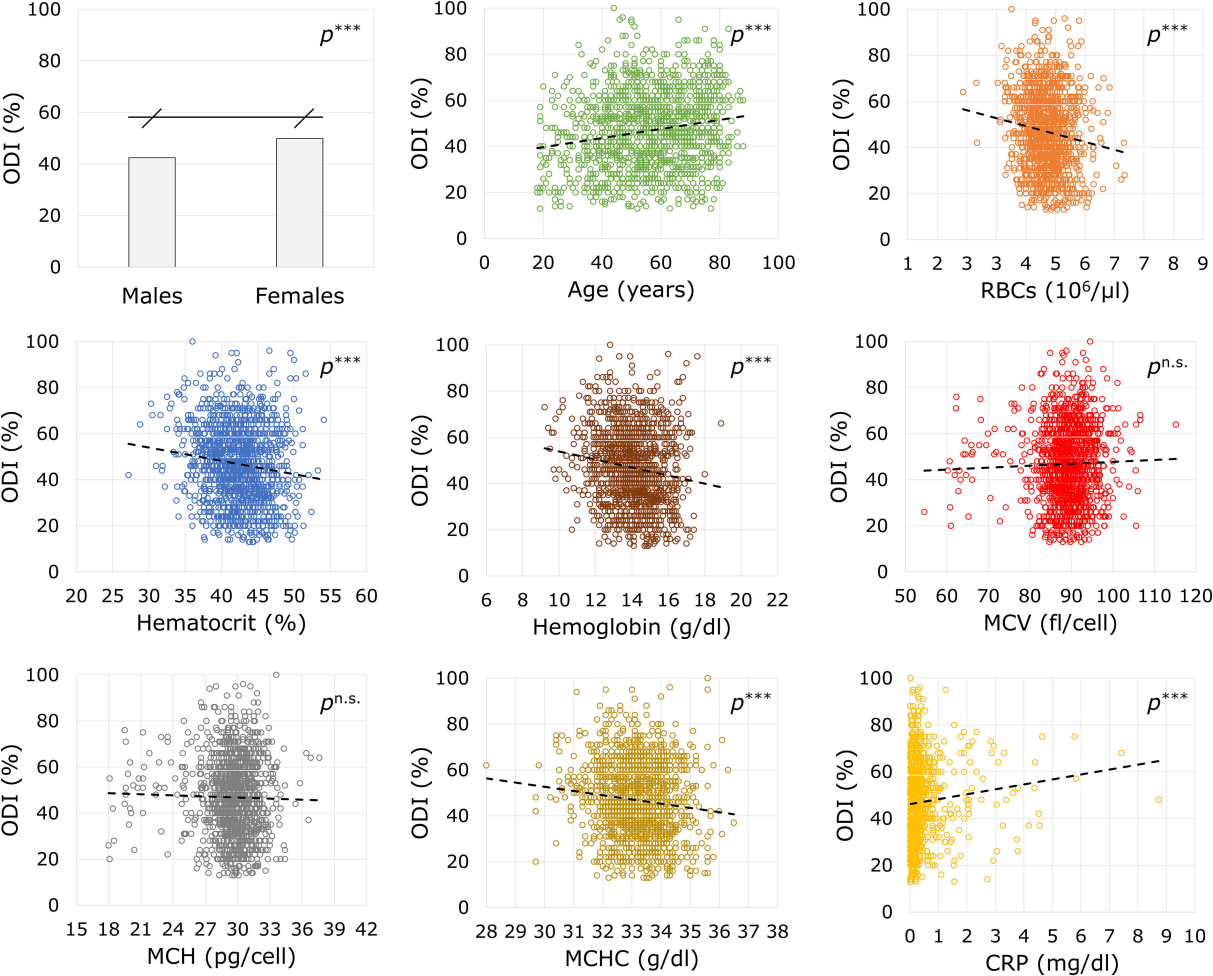
Associations between demographic and biochemical variables with the disability index before surgery. ODI, Oswestry Disability Index, ranging from 0 (no disability) to 100 (maximum disability); RBCs, red blood cells; MCV, mean corpuscular volume; MCH, mean corpuscular hemoglobin; MCHC, mean corpuscular hemoglobin concentration; CRP, C-reactive protein. Statistics: ***p < 0.001; ^n.s.^*p* not significant.

The association of sex, the years of age, and the biochemical markers with the ODI score at baseline are reported in Figure 1. There was found a positive association between the preoperative disability with the years of age (*B* = +0.197, *p* < 0.00001, 95% CI = +1.142: +0.253) and CRP (*B* = +10.985, *p* < 0.00001, 95% CI = +6.162: +15.807). An inverse association was observed for RBCs (*B* = −4.117, *p* < 0.00001, 95% CI = −5.931: −2.302), Ht (*B* = −0.579, *p* < 0.00001, 95% CI = −0.814: −0.343), Hb (*B* = −1.736, *p* < 0.00001, 95% CI = −2.355: −1.117), and MCHC (*B* = −1.829, *p* = 0.00003, 95% CI = −2.686: −0.972). No association were found with circulating MCV (*B* = 0.121, *p* = 0.219, 95% CI = −0.072: +0.314) or MCH (*B* = −0.176, *p* = 0.511, 95% CI = −0.701: +0.349). Men showed lower values of disability against women (42.42 ± 16.22% vs. 49.88 ± 15.86%; *U* = 172,804.50, *Z* = −8.294, *p* < 0.00001).

### Exploration of the Outcome

In the whole cohort, 1,022 patients recovered at least 30% from baseline ODI at 17 months, while the reduction of 12.7 points counted 1,019 successful outcomes. The recovery from disability considering 30% reduction showed an association with gender [χ_(1)_ = 5.339, *p* = 0.021], with about 76.72% of males and 71.15% of females encountering a recovery. However, the gender association was not confirmed [χ_(1)_ = 0.017, *p* = 0.895] after considering the reduction of 12.7 points. Patients with a unsuccessful recovery of at least 30% reduction were older at baseline (*U* = 156,017.50, *Z* = −4.990, *p* < 0.00001) with lower values of RBCs (*U* = 165,980.50, *Z* = −3.385, *p* = 0.001), Ht (*U* = 170331.50, *Z* = −2.828, *p* = 0.005), Hb (*U* = 163,994.50, *Z* = −3.786, *p* = 0.0001), MCHC (*U* = 158,815.00, *Z* = −4.521, *p* < 0.00001), but higher values of MCV (*U* = 164,810.00, *Z* = −2.603, *p* = 0.009). No differences for baseline MCH (*U* = 178,818.50, *Z* = −0.051, *p* = 0.960) and CRP (*U* = 130,854.50, *Z* = −1.711, *p* = 0.087). Considering the reduction of 12.7 points, the results were confirmed for age (*p* = 0.002), MCH (*p* = 0.920), MCHC (*p* = 0.026), and CRP (*p* = 0.540), but no differences were found for RBCs (*p* = 0.098), Ht (*p* = 0.122), Hb (*p* = 0.059), and MCV (*p* = 0.147). There was found a significant association between the 30% reduction and ASA [successful outcome in 81.07% with ASA 1, 72.55% with ASA 2, and 61.23% with ASA 3; χ_(2)_ = 36.381, *p* = 0.00002], diagnosis [successful outcome in 61.11% of spine deformities, 80.57% of disc disease, and 70.07% of back surgeries; χ_(2)_ = 36.381, *p* < 0.00001], DS (209.83 ± 137.15 vs. 248.13 ± 152.25 min; *U* = 156,271.50, *Z* = −4.339, *p* = 0.00001), and LOS (4.58 ± 3.35 vs. 5.53 ± 4.17 days; *U* = 155,719.00, *Z* = −4.077, *p* = 0.00004). Considering the reduction of 12.7 points, the results were confirmed for ASA (*p* = 0.016), diagnosis (*p* = 0.00001), DS (*p* = 0.002), and LOS (*p* = 0.005), with baseline ODI resulting different between groups (*p* < 0.00001).

#### Predictive Model PRE_BIO_

The values of RBCs, Ht, Hb, MCV, MCH, MCHC, and CRP were entered in the regression model to predict the variation of ODI at 17 months after surgery. The predictive model (*N* = 1,179) with the highest correlation was RBCs (*standardized B* = −0.071, *p* = 0.015, 95% CI = −5.857: −0.622). The model reported below significantly predicted the ODI variation [*F*_(1, 1177)_ = 5.895, *p* = 0.015, *R*^2^ = 0.005].


$$\text{PR}{\text{E}}_{\text{BIO}}\left(17\;\text{months}\right)\;=\;-9.121-3.239\;\left(\text{RBCs}\frac{10^{6}}{\text{μ}\text{l}}\right)$$

The estimated values calculated using the regression equation (−24.63 ± 1.53; min = −30.75; max = −18.48) showed no difference from real values (−24.37 ± 20.81; min = −96.00; max = +58.00) (*Z* = −0.079, *p* = 0.937). Using the 17-month reduction of 30% from baseline ODI score, increasing RBCs was associated with an increased likelihood of exhibiting a positive clinical outcome (*B* = 0.431, *p* = 0.001). The OR for having a positive outcome resulted in 1.539 (95% CI = 1.188: 1.992). The logistic regression model with RBCs correctly classified 73.34% of cases and was able to explain 1.14% (*Nagelkerke R*^2^) of the variance in the clinical outcome, with very low misspecification in its predictive capacity [Hosmer-Lemeshow test ${\text{χ}}_{\left(8\right)}^{2}$ = 13.207, *p* = 0.105]. The RBCs-based NN analysis using the progress of 30% showed 34.75, 20.00, 24.48% of incorrect predictions of the holdout phase. The models resulted to be a poor diagnostic instrument (first run AUC = 0.553; second run AUC = 0.565; third run AUC = 0.559). Using the reduction of 12.7 from baseline, increasing RBCs was not significantly associated with an increased likelihood of exhibiting a positive clinical outcome (*B* = 0.185, *p* = 0.152). The OR for having a positive outcome resulted in 1.203 (95% CI = 0.934: 1.550). The logistic regression model correctly classified 73.19% of cases and was able to explain 0.2% of the variance in the clinical outcome. The NN analyses using the variation of −12.7 points showed 27.70, 29.14, and 24.27% of incorrect predictions of the holdout phase. In conclusion, the RBCs-based PRE_BIO_(NN) resulted to be a poor diagnostic instrument (first run AUC = 0.527; second run AUC = 0.533; third run AUC = 0.528).

#### Predictive Model PERI_BIODEMCLI_

The preoperative biomarkers were used to build the model at 17 months together with the two demographic variables of age and gender and the five clinical variables of diagnosis, ASA, baseline ODI, DS, and LOS. The model that resulted statistically significant [*F*_(5, 1365)_ = 93.487, *p* < 0.00001, *R*^2^ = 0.255] included the following predictor-specific coefficients: Hb (*standardized B* = −0.067, *p* = 0.006, 95% CI = −1.710: −0.281), age (*standardized B* = +0.100, *p* = 0.0003, 95% CI = +0.063: +0.207), ASA (*standardized B* = +0.063, *p* = 0.024, 95% CI = +0.300: +4.230), baseline ODI score (*standardized B* = −0.500, *p* < 0.00001, 95% CI = −0.688: −0.570), and LOS (*standardized B* = +0.129, *p* < 0.00001, 95% CI = +0.698: +1.498). Gender (*p* = 0.088), RBCs (*p* = 0.698), Ht (*p* = 0.100), MCV (*p* = 0.839), MCH (*p* = 0.992), MCHC (*p* = 0.140), CRP (*p* = 0.083), diagnosis (*p* = 0.820), and DS (*p* = 0.265) were excepted.


$$\begin{array}{l} \text{PER}{\text{I}}_{\text{BIODEMCLI}}(17\;\text{months})\;=\;+2.021-\left[0.995\times \left(\text{Hb}\frac{\text{g}}{\text{dl}}\right)\right]\\ \;+\left[0.135\times \left(\text{years}\;\text{of}\;\text{age}\right)\right]\\ \;+\left[2.265\times \left(\text{ASA}\right)\right]\\ \;-\left[0.629\times \left(\text{preoperative}\;\text{ODI}\right)\right]\\ \;+\left[1.098\times \left(\text{days}\;\text{of}\;\text{hospital}\;\text{stay}\right)\right] \end{array}$$

However, the extreme distributions of the regression predicted values (−24.65 ± 10.42%; min = −64.55; max = +0.62) differenced from real values (*p* < 0.05) (−24.37 ± 20.81%; min = −96.00; max = +58.00). Using the reduction of 30% from baseline ODI score, a greater likelihood of exhibiting positive outcome was again associated with increasing Hb (*B* = 0.100, *p* = 0.035, OR = 1.106, 95% CI = 1.007: 1.213), decreasing age (*B* = −0.016, *p* = 0.001, OR = 0.984, 95% CI = 0.975: 0.994), and shorter LOS (*B* = −0.093, *p* = 0.0003, OR = 0.911, 95% CI = 0.866: 0.958). However, no association was found with the preoperative ODI score (*B* = 0.008, *p* = 0.051, OR = 1.008, 95% CI = 1.000: 1.013) and ASA (*B* = −0.196, *p* = 0.136, OR = 0.822, 95% CI = 0.636: 1.064). The logistic regression model correctly classified 73.67% of cases and was able to explain 5.39% (*Nagelkerke R*^2^) of the variance in the clinical outcome, with very low misspecification in its predictive capacity [Hosmer-Lemeshow test $\chi_{\left(8\right)}^{2}$ = 3.896, *p* = 0.866]. In Table 5 are reported the percentages of clinical success based on ranges of RBCs and Hb. There were expected 96.5% of true positives and 92.7% of false positives after setting a value of RBCs to 4 10^6^/μl. Similarly, setting a value of Hb to 12 g/dl exhibited about 93.8% of the positive outcomes correctly classified as positive, but 88.9% of the negative outcomes incorrectly specified as positive.

**Table 5:** Recovery from disability trends using scaled erythrocytes and hemoglobin.

**Predictors**	**Baseline ODI**	**17-month ODI**	**17-month success %**
**RBCs (10** ^**6**^ **/μl)**			
<4 (*n* = 63)	55.48 ± 16.41 (24.00; 100.00)	32.54 ± 21.67 (00.00; 86.00)	58.73%
4–4.49 (*n* = 327)	48.45 ± 15.85 (14.00; 95.00)	25.47 ± 20.38 (00.00; 93.00)	70.64%
4.5–4.49 (*n* = 606)	46.04 ± 15.76 (13.00; 95.00)	21.83 ± 19.20 (00.00; 86.00)	73.93%
5–5.49 (*n* = 300)	45.68 ± 17.00 (13.00; 96.00)	19.52 ± 19.07 (00.00; 97.00)	77.33%
≥5.50 (*n* = 96)	44.33 ± 18.15 (14.00; 95.00)	18.95 ± 20.65 (00.00; 86.00)	77.08%
**Hb (g/dl)**			
<12 (*n* = 87)	54.83 ± 14.82 (20.00; 84.00)	33.01 ± 23.78 (00.00; 86.00)	58.62%
12–12.9 (*n* = 195)	50.08 ± 16.57 (14.00; 100.00)	26.64 ± 19.92 (00.00; 93.00)	67.18%
13–13.9 (*n* = 373)	47.41 ± 15.22 (14.00; 95.00)	23.39 ± 19.00 (00.00; 80.00)	73.73%
14–14.9 (*n* = 380)	44.68 ± 15.69 (13.00; 91.00)	19.36 ± 18.88 (00.00; 97.00)	77.63%
15–15.9 (*n* = 237)	44.45 ± 16.83 (14.00; 88.00)	20.23 ± 19.31 (00.00; 90.00)	75.95%
≥16 (*n* = 120)	45.57 ± 19.38 (14.00; 96.00)	19.53 ± 20.29 (00.00; 78.00)	75.00%

*ODI, Oswestry Disability Index, ranging from 0 (no disability) to 100 (maximum disability); RBCs, red blood cells; Hb, hemoglobin. Clinical success, a 30% decrease from preoperative ODI at 17 months after surgery*.

Considering RBCs, Hb, the two demographic variables, and the five clinical variables, the PERI_BIODEMCLI_(NN) with 30% reduction showed 29.46, 22.63, 25.71% of incorrect predictions in the holdout phase. The model resulted to be a fair diagnostic instrument (first run AUC = 0.628; second run AUC = 0.632; third run AUC = 0.626), with normalized importance of 100.00% given by age in the first and third run and LOS in the second run. Using the 17-month progress of −12.7 from baseline ODI score, the odds were confirmed for age (*p* = 0.002), ASA (*p* = 0.154), and LOS (*p* = 0.001), but Hb was no more a significant predictor (*p* = 0.088) whereas baseline ODI significantly predicted the outcome (*p* < 0.00001). The logistic regression model correctly classified 74.91% of cases and was able to explain 10.81% of the variance in the clinical outcome. The PERI_BIODEMCLI_(NN) with 12.7 point reduction showed 34.69, 30.22, 29.17% of incorrect predictions in the holdout phase. The model resulted to be a fair diagnostic instrument (first run AUC = 0.720; second run AUC = 0.726; third run AUC = 0.723), with normalized importance of 100.00% given by baseline ODI.

## Discussion

**Figure 2:**
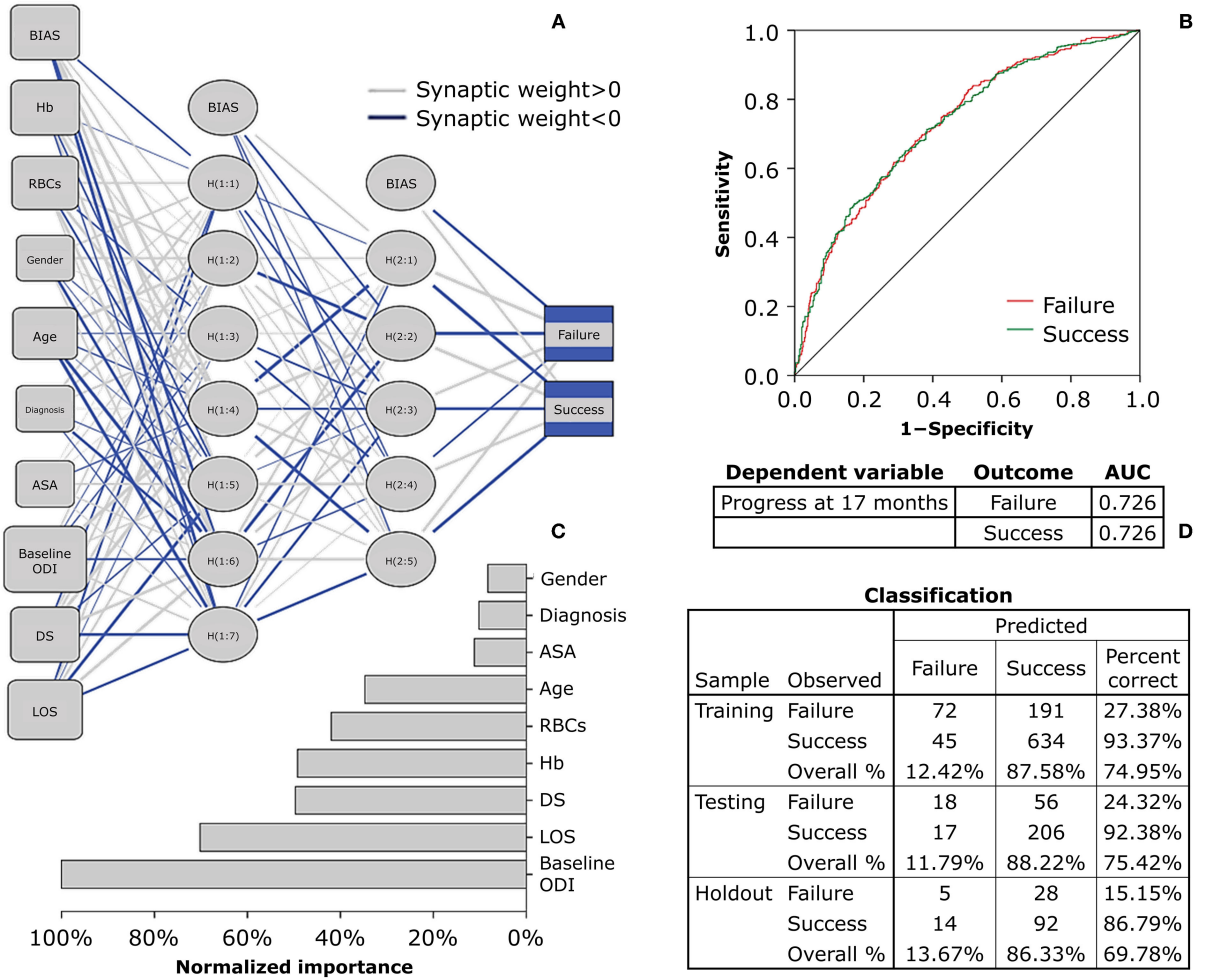
Multiplayer perceptron network predicting 17-month clinical recovery from disability using baseline biochemical, demographic, and clinical parameters. The Multilayer Perceptron Network (MPN) shows the prediction of 17-month recovery set as a raw decrease of 12.7 points from baseline ODI. **(A)** The architecture has two hidden layers, with the Sigmoid and Softmax being the hidden and output functions, respectively (criteria training = batch; optimization = scaled conjugate). **(B)** The network ROC curve (Receiver Operating Characteristic) shows a fair diagnostic performance of the parameters in predicting the last ODI score at 17 months after spine surgery. **(C)** The independent variables were rescaled (standardized) to show their normalized contribution in the model: gender (8.25%), diagnosis (10.20%), ASA (11.18%), age (34.72%), RBCs (41.95%), Hb (49.22%), DS (49.67%), LOS (70.20%), baseline ODI (100.00%). **(D)** The classification table of predicted vs. observed values was set with a training sample at 70%, a testing sample to track prediction at 20%, and a holdout sample to assess the final model at 10%. The final percent correct shows a high performance in predicting the clinically successful outcomes, but poor reliability to forecast negative outcomes. ODI, Oswestry Disability Index, ranging from 0 (no disability) to 100 (maximum disability); Hb, hemoglobin; RBCs, red blood cell count; ASA, American Society of Anesthesiologists physical status classification system (1, healthy; 2, mild; 3, severe; 4, life threatening; 5, moribund; 6, brain-dead); DS, duration of surgery; LOS, length of hospital stay.

It is foreseeable that the workload of spine surgery centers will intensify in the coming years as the population is getting older and debilitating polymorbid conditions are becoming not uncommon (19, 20). Technological advances in spine treatments help maintain short-term patient satisfaction high (21). However, it is necessary to plan patient-centered care pathways to achieve long-term results, thus revising the determinants of clinical success that simultaneously capture the perspective of the surgeon, the anesthesiologist, and the patient. In the present study, we analyzed the predictive potential of the preoperative biochemical markers on the ODI score at 17 months after surgery in patients enrolled in the institutional SpineReg of IRCCS Orthopedic Institute Galeazzi. The study cohort involved 1,392 patients undergoing surgery for deformity, disc disease, or other back spine disorders, and consisted of a majority of female older adults (Table 2). In absolute terms, an improvement in disability at the last follow-up was observed in over 88% of patients. Considering the more restrictive MCID of the ODI, about 73% of patients reported a successful recovery. Similar rates have already been observed in spine patients (5). There were no differences in recovery between males and females, but individuals who did not experience a clinical improvement were older at the time of surgery, had higher ASA, and lower ODI. Equally, these trends based on clinical determinants are in line with previous studies (22). Patients with spinal deformities experienced lower recovery rates than the other clusters of diagnosis, conceivably due to the greater surgical complexity that requires longer operative times and prolonged hospitalization (Table 3). Analyses of laboratory values confirmed that males generally have higher levels of RBCs and Hb than females and that there is a significant depletion with increasing age, feasibly mirroring iron supply discrepancies common in older adults. Similarly, the positive association of MCV and MCH with age would suggest an etiology from cobalamin or folate deficiency, which are known to cause macrocytic anemia in older individuals with poor strength of constitution (23, 24). This consideration was corroborated by increased disability and inflammation found in older patients (Figure 1). Based on available laboratory parameters, predictive modeling demonstrated that RBCs and Hb levels prior to surgery were the strongest determinants of clinical success at 17 months in all types of spine surgery. In particular, the univariate linear model explains 0.5 of the postoperative change in disability, with each unit increase in RBCs being associated up to 1.539 times the probability of clinical success. However, the corresponding neural network models showed poor diagnostic performance, having an erroneous prediction rate of up to 34.75% and an AUC of 0.565, making it unreliable in terms of sensitivity and specificity. Furthermore, only slight reductions in disability scores (−17 to −31 for RBCs) could be predicted. The prediction accuracy for poor outcomes did not improve after setting lower blood values, failing to identify both highly successful outcomes and worsening observed in 140 patients at 17 months. Therefore, it can be reasonably argued that stratification of patients based on univariate cut-offs may not be recommended and that studying laboratory biomarkers as continuous variables might be preferable (25, 26). In fact, the results in Table 5 showing a comparable trend between blood parameters and success rates give both RBCs and Hb a strong connotation that is also relevant for the patient. With the inclusion of clinical parameters, the variables in the multivariate linear models were able to explain ~25.1% at 17 months. The crude contribution thus accounts for both worsening and notable improvements in respect to the previous univariate model. Although the equation was not still adequate in the prediction of postoperative recovery, the neural network model at the last follow-ups showed the highest diagnostic performance even for the more restrictive MCID (AUC between 0.720 and 0.726 with up to 15.15% of correct prediction of negative outcomes), providing a decreasing order of importance of the preoperative determinants: ODI, LOS, DS, Hb, RBCs, age, ASA, diagnosis, gender (Figure 2). Thus, Hb seems to have a high predictive potential even greater than variables of demographic or clinical nature. The importance of preoperative Hb has already been studied in relation to complications in children, adults, and older adults undergoing spinal surgery (27–29), but it is unclear how it affects patients' long-term daily activities. It is plausible to think that the blood concentration reflects not only the strength of the patient's constitution (e.g., nutritional status) (8, 9, 30), but also the disease-specific weaknesses whose complications might consequently affect the daily activities of the patients (31). For example, there was found an inverse association between Hb levels and the number of patients reporting fatigue and shortness of breath (32). Whatever the connection, it is undeniable the recognition of the predictive potential that Hb has in the many surgical fields (33, 34). This study has limitations. Although patients admitted for complications were excluded from this research, information on intraoperative (e.g., transfusion-associated complications) or postoperative events that did not require access in our hospital was not accessible, thus possibly explaining the inability of the models to predict worsening. Furthermore, the study cohort might not represent the population of patients undergoing spinal surgery in our hospital, being indicative only of those who have agreed to participate in the registry over the years. While the completeness of the registry was satisfactory, missing data at predefined follow-ups reached 40% and may have provided some bias to the results. However, the models at 17 months were built on the scores at the last controls, thus making the results consistent (35). Lastly, although they can be estimated on the basis of surgical plan, both operative times and days of hospitalization are information available only after the intervention, which could undermine the preoperative nature of models.

In conclusion, our study sheds light on the role of preoperative Hb and RBCs in predicting long-term recovery reported by patients. Based on this research, values of RBCs <4 10^6^/μl and of Hb <12 g/dl in both genders may be associated with excessive rates of long-term failure after spine surgery from a patient's perspective. However, the model is not reliable in its current form and should be integrated with multidimensional variables of demographic, laboratory, and clinical nature to investigate further recovery determinants, such as body weight (36), the psychological distress (37), or the propensity for postoperative movement (38) and social participation (39). The ideal predictive model should have both high sensitivity and low false-positive rates. This is especially relevant when the consequence of not identifying patients at risk for negative outcomes could affect long-term daily activities. The performance of predictive models also varies according to the extent of recovery considered clinically relevant (17, 18), a concept that places the need to involve the patient in planning the treatment path, thus making the pre-admission visit more patient-centered. In the future, the correct stratification of individuals at risk will ensure opportunities to optimize patient's health in time for surgery, more affordable clinical care, and greater patient's satisfaction (16, 40, 41).

## Data Availability Statement

The original contributions presented in the study are included in the article/Supplementary Material, further inquiries can be directed to the corresponding author/s.

## Ethics Statement

Ethical review and approval was not required for the study on human participants in accordance with the local legislation and institutional requirements. Written informed consent for participation was not required for this study in accordance with the national legislation and the institutional requirements.

## Author Contributions

MB, PP, FL, TC, and ED conceived and designed the research. MB, FL, TC, and ED collected the data and managed the database. MB analyzed the data and wrote the first draft of the manuscript. PP, FL, TC, ED, PR, MP, LS, RB, MB-B, GB, and PB revised the first draft and contributed to the manuscript sections. GB and PB supervised the study. All authors contributed to the manuscript revision and approved the submitted version.

## Conflict of Interest

The authors declare that the research was conducted in the absence of any commercial or financial relationships that could be construed as a potential conflict of interest.

## Publisher's Note

All claims expressed in this article are solely those of the authors and do not necessarily represent those of their affiliated organizations, or those of the publisher, the editors and the reviewers. Any product that may be evaluated in this article, or claim that may be made by its manufacturer, is not guaranteed or endorsed by the publisher.
